# Exploring person-centred care in relation to resource utilization, resident quality of life and staff job strain – findings from the SWENIS study

**DOI:** 10.1186/s12877-020-01855-7

**Published:** 2020-11-11

**Authors:** Anders Sköldunger, Per-Olof Sandman, Annica Backman

**Affiliations:** 1 Department of Nursing, Umeå University, Vårdvetarhuset, 901 87 Umeå, Sweden; 2 Division of Neurogeriatrics, Karolinska Institutet, Huddinge, Sweden; 3 NVS, Department of Nursing, Karolinska Institutet, Huddinge, Sweden

**Keywords:** Person-centred care, Resource utilisation, Quality of life, Job strain

## Abstract

**Background:**

A critical challenge facing elderly care systems throughout the world is to meet the complex care needs of a growing population of older persons. Although person-centred care has been advocated as the “gold standard” and a key component of high-quality care, the significance of care utilisation in person-centred units as well as the impact of person-centred care on resident quality of life and staff job strain in nursing home care has yet to be explored.

The aim of this study was to explore person-centred care and its association to resource use, resident quality of life, and staff job strain.

**Design:**

A cross-sectional national survey.

**Methods:**

Data on 4831 residents and 3605 staff were collected by staff working in nursing homes in 35 randomly selected Swedish municipalities in 2014. Descriptive statistics and regression modelling were used to explore associations between person-centred care and resource use, resident quality of life, and staff job strain.

**Results:**

No association was found between person-centred care and resource use. Person-centred care was positively associated with resident quality of life and was negatively associated with staff perception of job strain.

**Conclusion:**

Person-centred care does not increase resource utilisation in nursing homes, but beneficially impacts resident quality of life and alleviates the care burden in terms job strain among staff.

## Background

Worldwide, a larger proportion of people are reaching old age as life expectancy continues to increase [1]. The rising number of older persons is leading to increased demands for the provision of nursing home care, which in turn poses great strain on public resources in Sweden, as well as globally [2, 3]. Nursing homes have a high prevalence of persons with cognitive impairment and dementia (50–84%) [4–6], and the major cost drivers in dementia care are costs of long-term institutional care in nursing homes and the costs of informal care [7].

The number of beds in municipal elderly care in Sweden has decreased from 120,000 to 90,000 between 2000 and 2010 [8] even though the proportion of older persons continues to grow. Swedish elderly care organisations comply with the Scandinavian model of publicly funded and essentially publicly produced care services, where the national policy specifies that older persons should be supported in living independently with a high quality of life and that older persons in need of care are entitled to high-quality care [9]. The Swedish elderly care system can be described as formalised care services that are regulated by Swedish law and are financed and in most cases provided by the public sector through municipal taxes and government grants. The total cost of elderly care in Sweden was SEK 95.9 billion, (approx. EUR 10.7 billion) in 2010, and of this about 3% of the cost was financed by patient charges [8]. New regulations and recommendations have emerged with expectations on the elderly care organisations to operate more efficiently while providing high care quality based on a person-centred philosophy [10–12]. Person-centred services are currently recommended as a global strategy to address the diverse and expanding care needs of an aging population [12].

The concept of person-centred care (PCC) has progressed significantly in research and practice and has dominated the research field over the last 20 years and is now considered the gold standard for the care of older people [13–16]. PCC is focused on moving beyond illnesses and dysfunction in a person and seeks to maintain personhood despite illness by using personal experiences to individualise the person’s care and their environment. PCC also involves creating a supportive social environment, prioritising relationships, seeing behaviour from the person’s perspective, involving relatives in care, and offering shared decision-making [13, 17–19].

PCC is expected to lead to improved well-being and reduced ill health [13, 17, 18, 20], and previous studies have shown that residents might benefit from this approach in terms of reduced agitation [21, 22]. However, only a few studies have explored PCC in relation to quality of life, and among the few studies that exist the findings are inconclusive. PCC has been associated with higher resident quality of life [23, 24], and the same was true in a person-centred intervention study [21]. However, contradictory findings have also been reported where a randomised controlled trial comparing PCC and “ordinary care” did not show any significant change in terms of quality of life among residents [25], nor did PCC interventions, the creation of more person-centred environments, or combinations of the two interventions [26, 27]. A systematic review with purpose to determine the effectiveness of organizational-level person-centred care for people living with dementia in relation to their quality of life showed no significant improvement in quality of life scores with person-centred care (intervention group), compared with usual care (control group). However, a significant effect in the quality of life score could be found within the person-centred care group (intervention group) [28]. This indicates that the findings in this research area are inconclusive and studies exploring how PCC is related to resident quality of life is still needed.

Given the continued worldwide societal costs of care due to aging populations [29] and PCC being advocated as a global strategy to address the diverse range of emerging care needs [12], it seems highly relevant to explore the relation between PCC and resource use. However, this has only been sparsely evaluated before, and among the few studies that exist, PCC has been associated with increased resource use [30], but also with better time efficiency [31, 32]. Implementing person-centred interventions has also been associated with increased costs related to staff education and training during the intervention phase. However, implementation costs of the training programme for PCC were lower than for dementia-care mapping, indicating that PCC might be a more cost effective care model, at least in the implementation phase [22]. Also, if hypothesising that PCC increases resource use (i.e. it increases the direct care hours provided to a resident), this might have an immediate effect on the level of job demands for staff working conditions under prevailing fiscal restraints. The combination of high job demands and low job control in the work environment is postulated to predict job strain [33]. Because elderly care staff are known to have a challenging and demanding job in terms of job strain, [34–39] and because they are exposed to increased risk for adverse health effects [40–43], it seems relevant to explore the association between PCC and resource use. Job strain is also a precursor to burnout, a related psychological syndrome of emotional exhaustion, depersonalisation, and reduced personal accomplishment. Burnout develops as a response to prolonged work stressors when the work demands exceed the capacity of the individual to deal with them [44]. Various person-centred interventions have reported that PCC might reduce staff exhaustion and burnout [45–47], thus highligting the significance for staff health to work in a person-centred manner. It has also been suggested that addressing staff stress and strain are an important focus when striving to improve PCC [48]. This is supported by a recent Swedish study by Sjögren et al. [49] that showed that higher levels of job strain are related to lower levels of PCC, indicating that perceived job strain might also impact the provision of PCC. Based on this, it seems important to explore how job strain is related to PCC in everyday practice, especially given the major impact this might have on staff and resident health in nursing home care. Thus, the presence of strain might hamper staff efforts to address PCC as the philosophy of care. To summarise, PCC has been advocated as the “gold standard” and a key component of high quality of care and has been advocated as a global strategy to address the diverse range of emerging care needs. However, the putative disparities in resource utilisation in person-centred vs. less person-centred units and the impact of PCC on resident quality of life and staff job strain in nursing home care has yet to be explored. Thus, the aim of this work was to explore PCC and its association with resource use, resident quality of life, and staff job strain.

## Methods

### Design

The Swedish National Inventory of Care and Health in Residential Aged Care (SWENIS) is a large, ongoing longitudinal study with explorative design conducted within the Umeå Aging and Health Research programme [45] and this study was based on SWENIS baseline data. Ethical approval for this study was obtained from the Regional Ethical Review Board in Umeå (Dnr 2013–269-31).

### Sampling and data collection

In Sweden a municipality is an administrative unit which is responsible for the community service including among other things schools, social care and care for the elderly. Out of 290 municipalities in Sweden, 60 municipalities were randomly selected and invited to participate, and 35 agreed to participate. The randomisation was done by letting a computer randomly select 60 of 290 municipalities. Thereafter, the managers in the nursing homes within these municipalities were contacted by telephone, inviting the nursing home to participate. Nursing homes which accepted the invitation got surveys sent to the nursing home, both to staff and to residents by mail. The data for this study were collected through two different surveys: (a) a self-reporting staff survey comprising demographic variables and established questionnaires for assessing perceived PCC (Person-centred Care Assessment Tool (P-CAT)) and job strain (The Swedish Demand-Control-Support Questionnaire (DCSQ)) and (b) a residents survey giving data on resident characteristics, health-related quality of life, and functional and cognitive status. Assessments of residents were performed by the staff member who knew each resident best (proxy rating). A total of 5423 staff surveys were distributed, and 3605 were returned (response rate 66.5%) from 169 nursing homes. Of 6902 resident surveys that were sent out, 4831 were returned (response rate 70%) from 172 nursing homes. The data collection took place from November 2013 to September 2014.

### Study context

In Sweden, there are approximately 2300 nursing homes with approximately 82,000 residents [46]. Swedish nursing homes are defined as housing for the elderly aged over 65 years who are no longer able to live at home due to extensive personal care needs and/or cognitive impairment [46]. Participating units included both special care units for persons with dementia (31%) and general units caring for older people (69%), and the number of beds in the facilities varied between 7 and 128 beds (median 38 beds). Most of the nursing homes in this sample (93.5%) were publically managed, and the remaining 6.5% were run by private providers.

### Study variables

#### Demographic variables

Demographic data included self-reported data on staff characteristics (i.e. sex, age, qualifications, work experience, and shift work) and proxy-rated data on resident characteristics (i.e. sex, age). An adapted version of the Katz activities of daily living was used to assess resident functional ability [50]. It measures the ability to manage ADL independently in six domains: bathing, dressing, transferring, toileting, eating, and continence. The modified version contained, for each domain, dichotomous scores, where functional dependency was scored “0” and fully functional independency was scored “1” which results in a total score of 0 to 6 points. Higher score indicate greater independence. Cognitive status was assessed using a scale developed by Gottfries and Gottfries (1968) consisting of 27 items regarding ability to orientate. Statements are answered with a “yes” [1] or “no” (0) resulting in a possible score of 0 to 27 where a higher score indicate better cognitive status, and scores < 24 indicate cognitive impairment [51].

#### Person-centred care assessment tool (P-CAT)

The P-CAT is a self-reported instrument [52, 53] used to assess to which extent the staff perceive the care provided to be person-centred. The Swedish version is a five-point Likert-type scale including 13 items (1 = disagree completely; 5 = agree completely). A total score can be calculated with a possible range of 13–65. A mean score per elderly care unit was calculated, and if a unit was missing data a facility mean was imputed for that unit. Higher scores indicated higher levels of PCC.

#### The Swedish demand-control-support questionnaire (DCSQ)

Two subscales of the DCSQ were used to measure the extent to which staff experience job strain. The used subscales was self-reported psychological demands and decision latitude (control) from the DCSQ [33]. The subscales consist of a four-point Likert-type scale where staff are asked to rate their perception of their job strain (1 = no, never; 4 = yes, often). The psychological demands consist of five items and decision latitude of six items. The subscales were used to calculate the dependent continuous variable of job strain by dividing the sum of psychological demands by the sum of the decision latitude (control) (cf. 39). The job strain index has a range of 0.21–3.33, with a higher value indicating greater strain [39].

#### Resource utilization in dementia (RUD)

Staff time for caregiving was measured by staff by using the RUD instrument [54], which is a comprehensive instrument for the assessment of direct care activities. The RUD comprises three domains – basic ADL, instrumental ADL, and supervision – and time is registered in hours and minutes during an ordinary day with a time frame working backwards from the last week. The Swedish version of the RUD has shown satisfactory estimates of reliability and construct validity in both on formal and informal care settings [55–58].

#### Health related quality of life (EQ-5D)

The EQ-5D is a generic quality of life and health status instrument designed by the EuroQoL group. The EQ-5D comprises five dimensions – mobility, self-care, usual activities, pain/discomfort, and anxiety/depression. Each dimension has three levels of perceived problems: 1 = no problems, 2 = some problems, and 3 = severe problems. The EQ-5D index scores were calculated using preference scores generated from a population using the EQ-5D-5L_Crosswalk_Index_Value_Calculator.v2 (Value Health, 2012) using the Danish tariff because Swedish values have not been calculated. This tariff was used to calculate a utility index for each resident. EQ-5D index scores range from 0 to 1, where 0 indicates the worst possible health state and 1 indicates the best possible health state.

### Data analysis

The IBM SPSS statistics version 25 was used to analyse the data. The scale distribution and normality of the scales were tested through measures of skewness, Kolmogorov–Smirnov analysis, and visual examination of the histograms. Missing items in the P-CAT were replaced with the mean values of the individual for the total scale, and up to two missing items were replaced (< 8%). For the two subscales of the DCSQ, only one item was replaced – Psychological Demands (< 3.4%) or Decision Latitude (< 2.2%) (cf. 56). No violations of the prerequisites for linear regression concerning multicollinearity normality, linearity, or homoscedasticity of residuals were detected (cf. Pallant 2013, p. 164). A *P*-value < 0.05 was considered statistically significant. All analyses of PCC were based on an aggregated unit-level score (mean value for the unit), while all resident and staff variables were based on individual scores.

First, descriptive statistics were used for background characteristics of staff and residents. Second, Pearson’s product-moment correlation was used to explore the correlation between PCC, staff job strain, quality of life, resident quality of life, and resource use. Third, a multiple linear regression analysis was conducted to explore the relationship between PCC, staff job strain, resident quality of life, and resource use. The model was adjusted for resident cognitive status, age, dependence in ADL, and sex.

## Results

### Characteristics of the sample

The sample of 3605 staff had a mean age of 46.6 years (SD 11.3 years) and comprised mostly women (95.3%). The most common qualification among staff was enrolled nurse (82.5%), and the average length of work experience in the nursing home was 9.9 years (SD 8.0 years) (see Table 1).

**Table 1:** Background characteristics of staff *n* = 3605

	n ^a^ (%)	m (SD)
Age (Years)		46.6 (11.3)
Sex		
Men	167 (4.7)	
Women	3401 (94.3)	
Did not state	37 (1.0)	
Qualifications		
Registered nurses	12 (0.3)	
Enrolled nurses	2918 (80.9)	
Nurse’s assistants	463 (12.8)	
No formal qualifications	82 (2.3)	
Other education	60 (1.7)	
Did not state	70 (1.9)	
Years of experience in aged care		17.9 (10.3)^b^
Years in this nursing home unit		9.9 (8.0)^c^
Day/night staff		
Day shift	80 (2.2)	
Day and evening	3140 (87.1)	
Day, evening, night shift	318 (8.8)	
Did not state	67 (1.9)	

^a^ Missing data on age for 76 persons

^b^ Missing data on 132 persons

^c^ Missing data on 209 persons

The sample of 4831 residents had a mean age of 85.5 years (SD 7.8 years) and comprised mostly women (67.8%). Most residents were ADL dependent (84%) and cognitively impaired (66.6%). The majority of residents resided in general units (62.2%) (see Table 2). The mean time for proxy raters (staff) knowing the resident was 3.6 years.

**Table 2:** Demographics of residents *n* = 4831

	n (%) ^a^	m (SD)
Age (Years)		85.5 (7.8)^b^
Sex		
Men	1538 (31.8)	
Women	3239 (67.0)	
Missing data	54 (1.1)	
ADL Capacity		
Independent	716 (14.8)	
Dependent	3768 (78.0)	
Missing data	347 (7.2)	
Cognitive impairment		
Yes	2827 (58.2)	
No	1418 (29.4)	
Missing data	586 (12.1)	
Residing in SCU	1778 (37.8)	
Residing in general units	2931 (60.7)	
Missing data	122 (2.5)	
Length of stay in months		30.5 (33.1)

^a^% does not always add up to 100 in all variables due to roundings’

^b^ Missing data on age for 704 persons

### Relationship of PCC with resource use, quality of life, and job strain

No correlation was found between PCC and resource use (*r* = − 0.0.45, *p* = 0.10). There was a significant positive correlation between PCC and quality of life (*r* = 0.106, *p* < 0 .000) and a significant negative correlation (*r* = − 0.423, *p* < 0.001) between PCC and job strain. The mean values and correlations between all study variables are shown in Table 3.

**Table 3:** Mean values and correlations of study variables

	N	M (sd)	***r***	***r***	***r***
			PCC (P-CAT)	Resource use (RUD)	HRQoL (EQ5D)
PCC (P-CAT)	3554	50.0 (7.4)			
Resource use (RUD)	2360	40.7 (37.4)	0.029		
HRQoL (EQ5D)	3062	0.70 (0.08)	0.065^b^	−0.45^a^	
Job Strain (DCSQ)	3482	0.71 (0.17)	−0.563^b^	−0.023	− 0.66^b^

^a^ Correlation is significant at the 0.05 level (2-tailed). ^b^ Correlation is significant at the 0.01 level (2-tailed)

The multiple linear regression analysis constructed with PCC as the dependent variable showed that a higher degree of PCC was not significantly related to higher resource use (standardised β = 0.031, *p* < 0.129). A higher degree of PCC was significantly related to higher quality of life among residents (standardised β = 0.042, *p* < 0.028). Furthermore, a higher degree of PCC was also significantly related to a lower level of perceived job strain among staff (standardised β = − 0.53, *p* < 0.001). This model explained 35.3% of the variance of PCC (Table 4).

**Table 4:** Multiple linear regression model explaining the variance of person centred care^a,b^

Independent variables	Unstandardised Coefficients		Standardised Coefficients	***p***-value
	β	Std.Error	St. β	
HRQoL (EQ5D)	3.620	1.648	0.042	0.028
Resource Use	0.007	0.004	0.031	0.129
Job strain (staff)	−22.733	0.837	−0.525	0.000

Dependent Variable: Person-centred care (P-CAT)

Adjusted R Square 0.35

^a^ Model adjusted for potential confounders (resident age, sex, physical function (ADL) and resident cognitive status)

^b^ N for joint analysis 1787 persons due to missing values

## Discussion

The aim of this study was to explore PCC and its association with resource use, resident quality of life, and staff job strain. The findings indicate that increased PCC provision was not related to increased resource use, but a higher degree of PCC was related to higher resident quality of life and to lower staff job strain.

A reasonable interpretation of these results is that PCC provision does not require increased care hours for nursing home providers once PCC has been implemented and that many nursing interventions do not require more resources even though they are performed in a more person-centred manner. For example, it takes as much effort to apply cream to an elderly lady’s back in a way that makes her feel like a queen as it does to just apply the cream without reflecting on how it is done.

In Sweden, PCC is recommended by the National Board of Health and Welfare (NBHW) [10]. The NBHW estimated that PCC would increase the need for resources in Swedish elderly care when training for and implementing PCC, a cost increase that has been confirmed in previous intervention research [22]. The NBHW [10] also estimated that the increased costs will be maintained over time because the provision of PCC is expected to increase the need for resources also in the long run [10]. However, this does not seem to hold true because PCC was not related to changes in resource use in the current study. A study based on the same sample as the current study showed that caring for residents with ADL dependency and cognitive impairment was related to increased resource use [59], indicating that this study population is already associated with high resource use. The findings of the current study showed that high-quality PCC does not need extended resources, which suggests that PCC might be provided within prevailing elderly care budgets. Estimating resource use in nursing homes has seldom been performed because Swedish elderly care has always offered an all-inclusive concept, but our previous study reported this thoroughly [59]. This situation will probably remain the same, but nevertheless it is important to clarify whether the “gold standard” of nursing home care, PCC, influences resource use. This study provides a small but seemingly important piece to the puzzle because it targets the WHO’s recommendation of PCC as a global strategy by exploring whether PCC as the model of care is still viable during times of restricted resources and when facing future challenges.

The data also showed that higher PCC was associated with higher resident quality of life, which is supported by previous cross-sectional and intervention studies [21, 23, 24]. Although quality of life is not a measure of quality of care per se, it might serve as a proxy in this sense. One reasonable interpretation is that when staff are working in a person-centred manner, which includes supporting resident autonomy, this enhances residents’ independence and autonomy in terms of mobility, self-care, and usual activities, all of which are aspects that contribute to high quality of life. This might imply that efforts can be made to maintain autonomy while also preventing functional dependency of this frail population by providing care based on a person-centred philosophy. However, because contradictory findings have also been reported in intervention studies with no significant changes from PCC on quality of life among residents [25–27], the relationship between PCC and quality of life seems complex and further studies are recommended. Further, the association, although statistically significant, might be of limited clinical importance due to the weakness of the association.

The nursing home work environment have long been known to be inherently stressful and demanding for staff who daily confronts a variety of job stressors with high risk of job strain as result [34, 36–38] Experiencing job strain has been related to increased risk for other negative health effects, such as; headaches, insomnia, poor concentration, irritability and nervousness [42, 43] and also sleeping problem [40], Other studies have suggested that staff who do not have the resources needed, or who are unable to provide the care they desire to, are also at risk for strain and burnout [37, 59]. Mitigating the perception of job strain is therefore crucial to protect staff health and wellbeing and consequently resident health and well-being [59]. Previous studies have also shown decreased stress and strain among staff when working in a person-centred manner [45–47, 49] and a higher level of PCC was also empirically related to lower perception of staff job strain in this current study.This study confirms these international findings by reporting strong associations between job strain and PCC. The theoretical assumption of job strain postulates that when the content of the work is too extensive in relation to the time available, with little or no opportunities to decide what, when, or how the work will be performed, staff are at risk of suffering from undo strain [33]. A reasonable interpretation of our findings is that staff, in line with a person-centred philosophy, are encouraged to initiate, become involved in, and take ownership of care provision, thus having the opportunity to organise the content and structure of care themselves. This might enable a balance between demands and control in daily practice with reduced job strain as a result.

A previous review [60] has also reported that working in a person-centred manner also improves job satisfaction, and reduces turnover in addition to reduction of stress, which ultimately may influence the quality of care provided giving further implications. Job strain can be seen as a measure of quality of work life and previous research has suggested the quality of work life must be one of the highest prioritised goals of the institutions, due to its major impact on care standards [61]. Addressing staff stress and strain seems therefor crucial given the major impact this has on the care organisation, staff and resident health, and the care that is provided [62]. The findings of this study thus suggest that PCC might be one plausible way to reduce the experience of job strain in nursing homes, thus having implications for care providers.

The levels of PCC within this sample of nationwide data are consistent with previous measures of PCC (measured with the P-CAT) in Swedish nursing home care [49], but slightly higher than international nursing home studies that have used the P-CAT [20, 63, 64]. Because the results of this study are part of a larger project with a longitudinal design, these could be of value as a point of reference for future national and international research studies.

### Methodological considerations

The cross-sectional design of this study comes with limitations because the causal direction of these findings cannot be established. Longitudinal and interventional designs are therefore needed to further investigate causality. In this study, the assessment of residents was made by staff through proxy ratings, which might be less than ideal but can be, as in this case, the only solution when including participants with a high prevalence of cognitive impairments [65]. However, in this study the proxies had known the older person for a fairly long time, implying good knowledge of the resident being assessed. Another limitation of the study is that it is based on the Swedish elderly care system, which might differ from international organisational conditions and thereby affect the study’s generalisability. Nevertheless, because this study draws on extensive randomised data from a national sample of Swedish nursing homes, it seems reasonable to argue that the findings can be applied across different contexts and settings. All instruments have been checked for normality, and only the RUD data were slightly skewed, but linear regression is still considered to be robust as long as no violations to the assumptions of residuals exist, which is the case in our data [66]. For the joint regression analysis the number of analysable persons are quite smaller than the total number of persons included in the study. This might of course introduce selection bias and the results should be interpreted with sound caution.

## Conclusion

To conclude, PCC does not increase resource utilisation, but positively impacts resident quality of life and alleviates the care burden in terms of job strain among staff. It also seems reasonable to conclude that providing PCC can be seen not only as a societal investment, but also as a strategy to sustain and improve wellbeing among nursing home residents and staff.

## Data Availability

The dataset supporting this article and its conclusions cannot be shared because informed consent has not been obtained for this.

## Abbreviations

ADL: Activities of Daily Living
DCSQ: The Swedish Demand-Control-Support Questionnaire
EQ-5D: EuroQoL Health Related Quality of Life
P-CAT: Person-centred Care Assessment Tool
PCC: Person Centred Care
RUD: Resource Utilization in Dementia
SWENIS: The Swedish National Inventory of Care and Health in Residential Aged Care
